# Frontotemporal Lobe Degeneration as Origin of Scans Without Evidence of Dopaminergic Deficit

**DOI:** 10.3389/fneur.2018.00335

**Published:** 2018-05-24

**Authors:** Manuel Menéndez-González, Tania Álvarez-Avellón, José M. Salas-Pacheco, Benito de Celis-Alonso, Kathryn A. Wyman-Chick, Oscar Arias-Carrión

**Affiliations:** ^1^Servicio de Neurología, Hospital Universitario Central de Asturias, Oviedo, Spain; ^2^Instituto de Investigación Sanitaria del Principado de Asturias, Oviedo, Spain; ^3^Departamento de Morfología y Biología Funcional, Universidad de Oviedo, Oviedo, Spain; ^4^Departamento de Psicología, Universidad de Oviedo, Oviedo, Spain; ^5^Instituto de Investigación Científica, Universidad Juárez del Estado de Durango, Durango, México; ^6^Facultad de Ciencias Físico Matemáticas, Benemérita Universidad Autónoma de Puebla, Puebla, México; ^7^Regions Hospital/Health Partners Neuroscience Center, Saint Paul, MN, United States; ^8^Unidad de Trastornos del Movimiento y Sueño/Centro de Innovación Médica Aplicada, Hospital General Dr. Manuel Gea González, Ciudad de México, México

**Keywords:** scans without evidence of dopaminergic deficit, parkinsonism, dopamine transporter-single, photon emission computed tomography, presynaptic dopaminergic deficiency, frontotemporal lobar degeneration

## Abstract

The term scans without evidence of dopaminergic deficit (SWEDD) can be associated with any patient diagnosed at first with Parkinson’s disease but with a negative dopamine transporter-single photon emission computed tomography (DaTSPECT), which does not confirm the presynaptic dopaminergic deficiency. Therefore, an alternative diagnosis should be sought to support parkinsonism as a clinical diagnosis. Parkinsonism is a well-known manifestation of frontotemporal lobar degeneration (FTLD), particularly frequent in those with positive DaTSPECT. Here, we reinforce previous observations that parkinsonism can be present in FTLD patients with negative DaTSPECT and therefore, FTLD may account for a percentage of patients with SWEDD. We gather the clinical observations supporting this hypothesis and describe a case report illustrating this idea. Studies suggest the result of DaTSPECT in FTLD may depend on the neuropathology and clinical subtype. However, most studies do not provide a clinical description of the clinical subtype or pathological features making the association between subtypes of FTLD and DaTSPECT results impossible at the moment. Further studies correlating clinical, neuropsychological, neuroimaging, genetic, and pathology findings are needed to better understand parkinsonism in FTLD.

## Introduction

Parkinson’s disease (PD) is a common neurodegenerative disorder. It is characterized by progressive degeneration of dopaminergic neurons in the *pars compacta* of the substantia nigra and the loss of nerve terminals in the basal ganglia structures (1, 2). The dopaminergic system is one of the most studied neurochemical systems because damage to nigrostriatal neurons is the most critical component in the pathophysiology of PD (1, 3). Clinically it is manifested by the so-called “parkinsonian syndrome,” consisting of extrapyramidal signs, including bradykinesia and at least one of the following: 4–6 Hz rest tremor, muscular rigidity and postural instability. The term “parkinsonism” encompass some nosologic entities, besides PD, which are grouped by their shared clinical features but are separated by their different pathologies.

The role of nigrostriatal dopamine deficits in PD has been firmly established (4–6). In recent years, dopamine transporter-single photon emission computed tomography, named DaTSPECT, has been used to detect degeneration of presynaptic dopamine receptors in the nigrostriatal structures (6, 7). The active ingredient of [123I]FP-CIT SPECT is a cocaine analog, 123I-nortropane, labeled: *N*-u-fluoropropyl 2b-carbomethoxy-3b-(4-iodophenyl), also referred to as: ([123I]ioflupane). It binds to striatal presynaptic dopamine transporter (DaT) in animals and humans and helps visualize indirectly these neurons with SPECT. DaT is located on the plasma membrane, mainly of nerve terminals of dopaminergic neurons in the brain, particularly in the globus pallidus, cingulate cortex, amygdala, olfactory tubercle, and midbrain but specially in the striatum and nucleus accumbens. DaT reuptakes dopamine from the synaptic cleft into presynaptic neurons playing an important role in the buffering of this neurotransmitter (8).

Baseline DaT imaging has a very high negative predictive value for degenerative conditions affecting the nigrostriatal pathway. Only a negligible proportion of normal dopaminergic SPECT can be found in atypical parkinsonisms (9). The acronym SWEDDs (scans without evidence of dopaminergic deficits) (10), arose from the clinical trial literature of PD, in which patients were imaged with 18F-dopa PET or DaTSPECT to monitor disease progression, revealing that a substantial proportion of clinically diagnosed cases of PD had regular nuclear medicine scans (4–15%) and were, therefore, designated as SWEDDs (10, 11). Thus, the term SWEDDs can be associated with any patient diagnosed at first with PD but in which subsequent functional imaging did not confirm the presynaptic dopaminergic deficiency, although the use of the term SWEDD is itself controversial (9, 12).

The true etiology of the symptoms experienced by patients with SWEDD remains controversial, and it has been suggested that these individuals may represent a non-PD related movement disorder (10, 13, 14). When patients with SWEDD were initially discovered, it was hypothesized that they might be within a prodromal phase of PD (15). However, subsequent research has demonstrated significant differences between patients with dopamine-deficient scans and patients with SWEDD. Patients with SWEDD lack response to levodopa (16) and do not demonstrate deficits in olfaction as frequently as patients with dopamine-deficient PD (17). Patients with SWEDD also have more significant cardiovascular and thermoregulatory dysfunction, orthostatic hypotension, sleep disturbances, and higher frequencies of daytime sleepiness than dopamine-deficient PD patients (18, 19). Although, patients with SWEDD can present motor features similar to those which are dopamine-deficient (PD), previous longitudinal research suggests that patients with SWEDD do not demonstrate the progression of motor symptoms (20) and continue to have normal DaTSPECTs for up to 4 years after they are initially identified (19, 21, 22). In a 5-year follow-up study of 16 patients with SWEDD, only 2 patients demonstrated reduced dopamine uptake on DaTSPECTs, while 14 remained classified as SWEDD (11). These studies seem to indicate that individuals with SWEDD have a distinctly different disorder than dopamine-deficient PDs (14, 15, 18, 22). Moreover, patients with SWEDD and long-standing parkinsonism exhibit non-motor features that differ from those of patients with PD. SWEDD patients had worse mood and cardiovascular function and better olfactory function than PD patients, but remain similar to patients with SWEDD and alternative final diagnoses (19). In general, the term SWEDD is a useful approach for those patients with slow-progressing parkinsonism, with mild evolution compared with PD. Some frontotemporal lobar degeneration (FTLD) have parkinsonism and a mild clinical course, probably this subgroup could represent a minority percentage of SWEDD (23–26).

In a prospective study of our research group, 30 patients with hard-to-diagnose tremor and normal DaTSPECT were followed for 2 years (27). After the follow-up scan diagnosis was reached for 18 cases. The other 12 patients underwent a second DaTSPECT and were then followed for 12 additional months. After this, the clinical diagnosis was performed again. The final diagnoses included a list of different entities, including neurodegenerative and non-neurodegenerative disorders. However, for six patients diagnosis remained uncertain. Interestingly, these six patients developed cognitive impairment with outstanding frontal features. It was first speculated that although some patients with SWEDDs had, in fact, dystonic tremor or other well-known neurological conditions, it was conceivable that some patients might have suffered from a disorder that had not yet been described. It was speculated that some of these patients might have suffered a neurodegenerative process originated in the frontal cortex spreading to subcortical structures (versus the subcortical → cortical process present in PD). In this article, we present a case report illustrating this possibility and discuss the clinical, neuropsychological, genetics, and neuroimaging findings supporting the observation that FTLD is behind some cases of SWEDD.

## Case Report

Written informed consent was obtained from the patient for the publication of this case report. She is a 70-year-old woman who worked as a childcare worker and is now retired. She used to drink 30 g alcohol/day until 10 years ago and she still smokes 5 cigarettes/day. She was diagnosed with depressive syndrome 10 years ago. Currently, she is on Trazodone 100 mg/day and Clonazepam 0.5 mg/day. There are cases of PD (brothers) and depression (mother and maternal grandmother) among her family records.

She first visited neurology clinics 10 years ago with memory loss complaints. On that occasion, cognitive screening tests, blood tests, and CT scan were normal. Complaints were supposed to be in relation with a mood disorder, and no treatment was prescribed. She came again 5 years ago due to impairment of memory. She also complained about unbalanced gait, intentional tremor, and difficulty doing fine tasks. Caregivers referred social, behavioral changes, and personal care difficulties, even when she was still living on her own at that time.

Neurological examination showed hypomimia, mild rigidity in the four limbs, global bradykinesia, and unbalanced gait. There was a mild and mixed (rest and action) tremor affecting the four limbs. Her Mini-Mental Status Examination reflected deficits in memory, while her performance on the Frontal Assessment Battery was notable for impairment in inhibiting automatic responses, verbal fluency, and Trail making tests A & B. She also demonstrated deficits in the memory and verbal fluency sections of the Seven Minute Screen.

**Figure 1:**
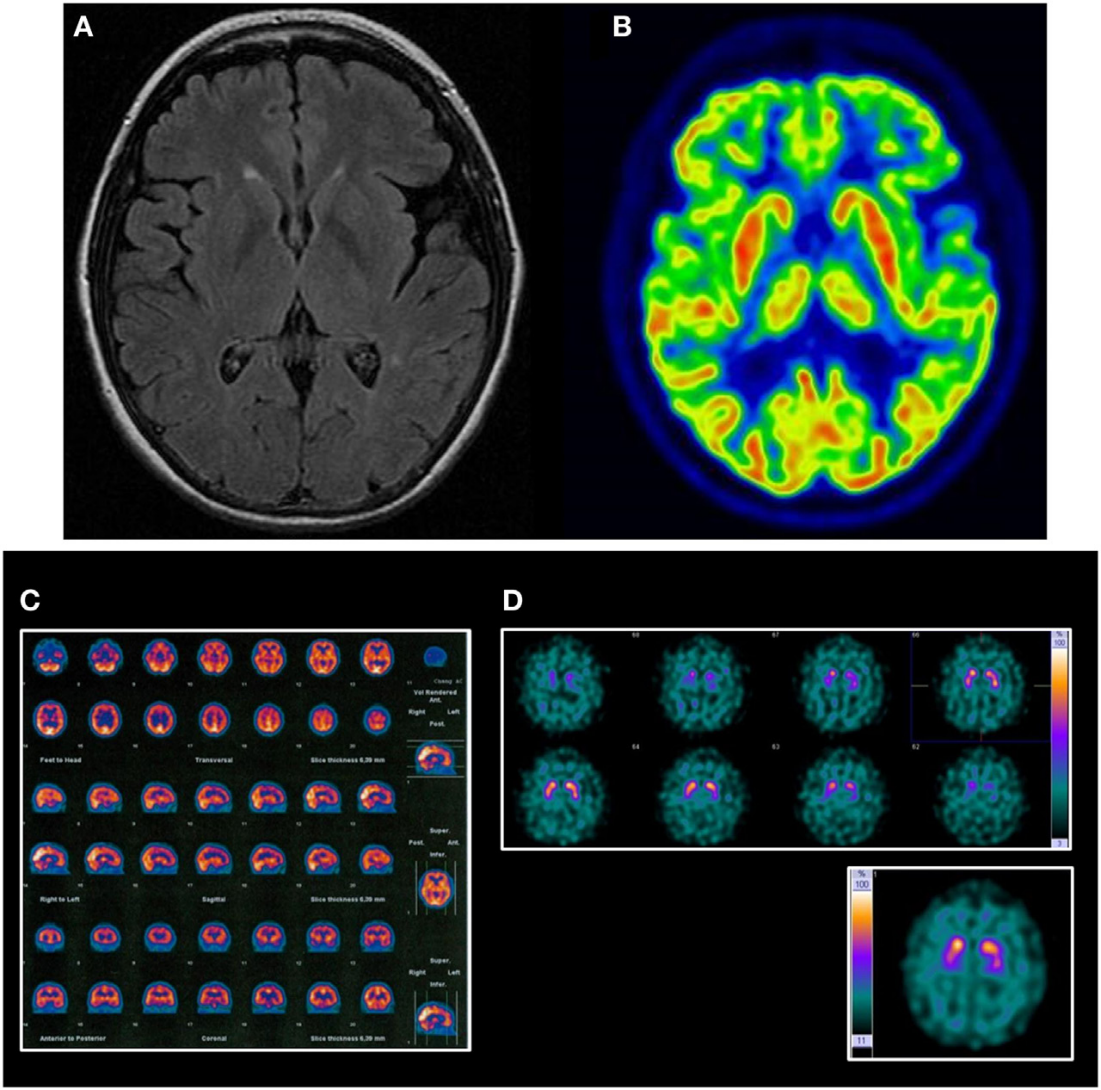
Neuroimaging findings in the reported case. **(A)** Follow-up MRI mild diffuse atrophy with more intense atrophy on the left frontal and temporal lobes and some small vessel lesions affecting periventricular and semioval white matter. **(B)** FDG-PET shows mild-to-severe hypometabolism on the left frontotemporal lobe junction and mild hypometabolism on the left fronto-basal, left anterior cingulum regions. **(C)** HMPAO SPECT shows bilateral but asymmetric hypoperfusion (more on the left side) on frontotemporal lobes. **(D)** Dopamine transporter-single, photon emission computed tomography was informed of the normal density ofpresynaptic dopaminergic uptakers. Some irregularities in the morphology of basal ganglia and increase of cortical uptake can be noted.

Laboratory tests were all normal. Baseline MRI showed mild diffuse atrophy of the cerebrum together with some small vessel lesions affecting periventricular and semioval white matter. Follow-up MRI (5 years later) showed more intense atrophy in the left frontal and temporal lobes (Figure 1A). Tc-99m-HMPAO SPECT showed bilateral but asymmetric hypoperfusion (more on the left side) on frontotemporal lobes (Figure 1B). 18F-FDG-PET showed mild to severe hypometabolism on the left frontotemporal lobe junction, and mild hypometabolism on the left fronto-basal left anterior cingulum regions (Figure 1C). DaTSPECT was informed as for the normal density of presynaptic dopaminergic uptakers (Figure 1D). Some irregularities in the morphology of basal ganglia and increase of cortical uptake were present.

The final diagnosis was frontotemporal lobe dementia—behavioral variant—with parkinsonism. Even when the density of presynaptic dopaminergic uptakers in the DaTSPECT was normal, she was put on Levodopa without significative clinical changes.

## Perspective

### Clinical Observations

Parkinsonism is a well-known manifestation of FTLD (28). To date, presentations of FTLD with motor or movement disorders include (1) frontal lobe degeneration (FTD) with motor neuron disease (FTD-MND), (2) corticobasal degeneration (CBD), and (3) progressive supranuclear palsy (PSP). CBS and PSP usually show parkinsonism. However, parkinsonism has also been reported as a relative finding in other subtypes of FTD apart from CBS and PSP. Indeed, parkinsonism is found in approximately 20–30% of patients with FTLD. Furthermore, parkinsonism can be seen in all FTLD subtypes, and it can even be an outstanding feature in some cases. Therefore, there is a need to investigate parkinsonism in FTLD to obtain a better understanding of the disease.

Regarding the clinical characteristics, features of parkinsonism in FTLD are variable. The classical akinetic-rigid syndrome usually characterizes it. Other cases show atypical parkinsonism resembling PSP or CBD. Although rare, parkinsonism in FTLD may coexist with MND. Parkinsonism in FTLD is usually levodopa unresponsive, but there have been cases where a temporary benefit has been reported. The lack of response to levodopa reinforces the idea of a pathogenic mechanism different to PD.

### Genetics

A systematic review aimed at the characterization of movement disorder phenomenology in genetically proven familial FTLD, showed that at any point during the disease, parkinsonism was the most common movement syndrome. It was reported in 79.8% of cases, followed by PSP and CBD syndromes in 12.2 and 10.7% of cases, respectively (28). The amyotrophic lateral sclerosis/parkinsonism dementia complex of Guam was probably the first known association of parkinsonism with dementia of frontotemporal features (29). Parkinsonism was frequently observed in familial FTLD, more specifically in FTLD with parkinsonism linked to chromosome 17q (FTDP-17) (30). Parkinsonism in familial FTLD was first described in families with mutations in the microtubule-associated protein tau (MAPT) and progranulin (PRGN) genes. Since then, mutations in several other genes have been identified for FTLD with parkinsonism, including chromatin modifying protein 2B, chromosome 9 open reading frame 72 (C9ORF72), fused in sarcoma, valosin-containing protein, and transactive DNA-binding protein (TARDBP) (31, 32). Mutations in seven genes were robustly associated with autosomal dominant (SNCA, LRRK2, EIF4G1, VPS35) or recessive (parkin/PARK2, PINK1, DJ1/PARK7) PD or parkinsonism (33, 34). Changes in a long list of additional genes have also been suggested as causes for parkinsonism or PD, including genes for hereditary ataxias (ATXN2, ATXN3, FMR1), frontotemporal dementia (C9ORF72, GRN, MAPT, TARDBP), DYT5 (GCH1, TH, SPR), and others (ATP13A2, CSF1R, DNAJC6, FBXO, GIGYF2, HTRA2, PLA2G6, POLG, SPG11, UCHL1) (34–37).

### Neuropsychology and DaTSPECT Imaging in PD and FTLD

Cognitive deficits could be identified in around a third of patients, even in the early untreated stages of PD (38–41). This cognitive dysfunction may have been related, in part, to reduced dopamine levels. While there were some inconsistent findings in the literature (42), fMRI studies with patients on and off levodopa indicated that higher levels of dopamine were associated with better cognitive performances on tasks of working memory and response accuracy (43). In addition, recent fMRI and DaTSPECT research have demonstrated a positive correlation between nigrostriatal dopaminergic function and performance on tests of executive functioning and memory (44). However, dopamine deficiency may not explain all of the cognitive deficits in PD, as some degree of cognitive impairment was common in patients diagnosed with related movement disorders such as dystonia (45) and essential tremor (46), conditions that were not associated with dopaminergic deficiency on imaging (13, 27, 36). These patients usually had an abnormal DaTSPECT as the damage in frontostriatal neural circuitry occurred down (basal ganglia) to up (frontal cortex) (47).

It is a well-known fact that cognitive impairment in early PD and other synucleinopathies (Parkinson-plus syndromes) was accompanied by reductions in activity in frontostriatal neural circuitry (48, 49). There were five fronto-subcortical circuits linking different regions of the frontal cortex and subcortical nuclei, involving several neurotransmitters. Nigrostriatal dopaminergic neurons played an important role in some of these circuits, but gabaergic, glutamatergic, and cholinergic neurons were also extensively represented. Therefore, neurodegenerative disorders affecting neurons outside the nigrostriatal dopaminergic circuits were not be detected by DaTSPECTs and would have still been able to produce extrapyramidal symptoms.

When dementia preceded PD, international consensus recommended a diagnosis of these patients with Dementia with Lewy bodies (DLB). Nevertheless, most patients with DLB and extrapyramidal signs had abnormal DaTSPECTs. In fact, autopsy studies provided class I evidence of 123I-FP-CIT dopaminergic neuroimaging accurately identifying patients with DLB (50), although this could be normal in those without extrapyramidal signs.

What is most relevant to the hypothesis presented in this paper is the fact that many SWEDD patients exhibited cognitive dysfunction. Studies with data from the Parkinson’s Progression Marker Initiative (PPMI) database, showed that SWEDD patients performed significantly worse in semantic fluency and processing speed when compared with healthy controls (51). Montreal Cognitive Assessment test (MoCA) scores showed that about one-third of the PPMI participants were clinically diagnosed with SWEDD, experienced a statistically significant cognitive decline over the relatively brief period of 2 years, and a higher proportion of participants with SWEDD than DaTSPECT-confirmed PD had cognitive decline. The participants in the SWEDD group were more than twice as likely (relative risk = 2.07) than those in the dopamine-deficient group to fall below the MoCA cognitive impairment cutoff. Thus, recently diagnosed patients with SWEDD may have been an even more significant risk for cognitive decline than patients with DaTSPECT-confirmed early-stage PD (52, 53). Neuropsychological performances between the SWEDD and the PD resulted similar when comparing patients in all stages (54). Altogether, these results suggested that SWEDD should not be considered a benign finding, as a high proportion of patients could be expected to suffer a cognitive decline in the close future.

Some studies have shown decreased uptake of DaT in bilateral putamina in FTLD with a negative correlation between the uptake ratio and parkinsonian motor status (55). Another study by Morgan and colleagues found that around a third of the FTLD cases may have presented abnormal scan and a significant reduction in uptake in the putamen and the caudate (56). Therefore, patients with FTLD may have had a positive or a negative DaTSPECT. This result probably depends on the neuropathology and clinical subtype of FTLD.

For instance, a study with patients with non-fluent/agrammatic variant of primary progressive aphasia (nfvPPA) and a logopenic variant of PPA (lvPPA) without clinical parkinsonism at baseline, showed reduced striatal tracer uptake in nfvPPA patients prior to clinical parkinsonism, especially for those nfvPPA without AD biomarkers, suggesting subclinical nigrostriatal degeneration (57). All lvPPA patients had normal DaTSPECT. Patients with nfvPPA presented abnormal DaTSPECT, especially in the left hemisphere compared with controls. DaTSPECT in nfvPPA patients with normal progranulin and negative CSF AD biomarkers was also significantly reduced compared with lvPPA patients with positive CSF AD biomarkers, suggesting nigrostriatal degeneration does not usually appear in the context of AD pathology resent, but it can appear in the context of FTD pathologies. During follow-up, seven nfvPPA/bio-patients developed parkinsonism, six of them with baseline reduced 123I-FP-CIT uptake (57).

## Final Remarks

The literature shows many clinical, genetic, and neuropsychological studies linking parkinsonism and FTLD. Also, a good number of articles provide evidence of the distinct characteristics between PD and SWEDD, and on the cognitive impairment present in many SWEDD cases. Finally, DaTSPECT studies in FTLD found both positive and negative results.

We report the case of a patient diagnosed with a behavioral variant of frontotemporal lobe dementia who also has parkinsonism that was not attributable to vascular lesions, antidopaminergic drugs, or any other etiology. DaTSPECT was informed as normal, and therefore it might be considered SWEDD.

On this basis, we reinforce the observation that parkinsonism without evidence of nigrostriatal dopaminergic deficit can be present in FTLD patients. Even when most of them will also present cognitive impairment, parkinsonism may be the main reason for consultation, and a normal DaTSCAN can be found during the diagnostic process. Therefore, FTLD may account for a percentage of patients with SWEDD. This origin might contribute to the observation that SWEDD patients exhibit a higher risk of cognitive impairment, worse mood, and better olfaction than PD patients (19, 51). Other causes of SWEDD have been described, particularly dystonic tremor. Thus, SWEDD should be understood as a heterogeneous group of patients in which some cases (probably the most tremoric ones) may have dystonic tremor, and some others may have FTLD (probably those with outstanding frontal cognitive dysfunction).

Careful clinical assessment remains the cornerstone of the study of patients with FTLD with parkinsonism. However, rationale support of genetic studies, neuropsychological evaluation, and neuroimaging, including structural and functional techniques, will provide a more detailed and comprehensive picture.

## Ethics Statement

Written informed consent was obtained from the patient for the publication of this case report.

## Author Contributions

All authors contributed equally.

## Conflict of Interest Statement

The authors declare that the research was conducted in the absence of any commercial or financial relationships that could be construed as a potential conflict of interest.
